# Characterization of deep neural network features by decodability from human brain activity

**DOI:** 10.1038/sdata.2019.12

**Published:** 2019-02-12

**Authors:** Tomoyasu Horikawa, Shuntaro C. Aoki, Mitsuaki Tsukamoto, Yukiyasu Kamitani

**Affiliations:** 1 ATR Computational Neuroscience Laboratories, 2-2-2 Hikaridai, Seika, Soraku, Kyoto 619-0288, Japan; 2 Graduate School of Informatics, Kyoto University, Yoshida-honmachi, Sakyo-ku, Kyoto 606-8501, Japan

**Keywords:** Visual system, Computational neuroscience, Functional magnetic resonance imaging

## Abstract

Achievements of near human-level performance in object recognition by deep neural networks (DNNs) have triggered a flood of comparative studies between the brain and DNNs. Using a DNN as a proxy for hierarchical visual representations, our recent study found that human brain activity patterns measured by functional magnetic resonance imaging (fMRI) can be decoded (translated) into DNN feature values given the same inputs. However, not all DNN features are equally decoded, indicating a gap between the DNN and human vision. Here, we present a dataset derived from DNN feature decoding analyses, which includes fMRI signals of five human subjects during image viewing, decoded feature values of DNNs (AlexNet and VGG19), and decoding accuracies of individual DNN features with their rankings. The decoding accuracies of individual features were highly correlated between subjects, suggesting the systematic differences between the brain and DNNs. We hope the present dataset will contribute to revealing the gap between the brain and DNNs and provide an opportunity to make use of the decoded features for further applications.

## Background & Summary

Building models that achieve human-level performance has motivated researchers to construct computational models that mimic the architectural and representational properties of the human brain. Adopting the hierarchical architecture of the human visual system, deep neural networks (DNNs) have demonstrated utility in various applications, including in object recognition in computer vision, where near human-level performances are achieved. This achievement has led to many comparative studies on the similarities between the brain and DNNs, providing empirical support for the correspondence between the hierarchical representations of the brain and DNNs^1–8^.

On the basis of the hierarchical representational similarity between the brain and DNNs, our recent study demonstrated that human brain activity measured by functional magnetic resonance imaging (fMRI) can be decoded (translated) into DNN feature values^6^. Combining such decoded DNN features and techniques developed with DNNs, recent work has started to develop new technologies to read out richer contents in the brain as demonstrated in the generic decoding of seen, imagined, and dreamed objects^6,7^, and in the reconstruction of seen and imagined images^9^. As exemplified by these studies, the decoding of DNN features from brain activity patterns can provide opportunities to develop new technologies for further applications in brain-machine interfacing.

In addition to the capability of the DNN feature decoding approach as a generative model of DNN signal patterns from the brain, the decoding approach also has the advantage of allowing the characterization of individual DNN units in terms of their decodability from brain activity patterns. Our decoding analysis of DNN features showed that not all DNN feature units were equally decoded^6^, indicating a gap between the DNN and human vision. Thus, evaluating the decodability of individual DNN units will help to further elucidate finer levels of representational similarity between the brain and DNNs, enabling the selection of highly decodable features for further analyses^10^.

**Figure 1:**
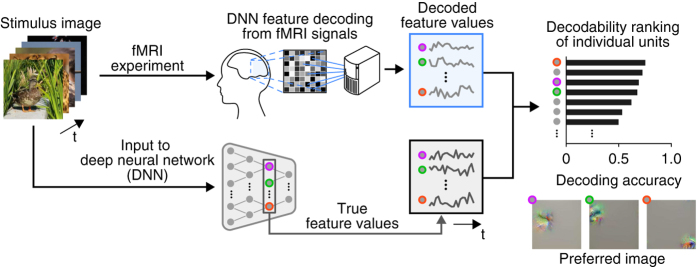
Overview of the data generation procedures. Stimulus images were presented to human subjects in the fMRI experiments to collect fMRI signals. DNN feature decoders were first trained to decode DNN feature values of presented images from the training fMRI data, and were then applied to test fMRI data to produce sequences of decoded feature values for all DNN units. The same stimulus images were also provided to DNNs as inputs and sequences of DNN feature values were computed for all DNN units. For each individual DNN unit, the decoding accuracy (or “decodability”) was evaluated using a Pearson correlation coefficient between the sequences of decoded and true feature values. The estimated decodability was used to rank the DNN units within each DNN layer. Examples of the preferred image of high-ranking units are shown at the bottom-right.

In this report, we present a dataset derived from the DNN feature decoding analyses from human brain activity patterns (Fig. 1). The dataset comprises fMRI signals measured while subjects viewed natural images (Data Citations 1, 2,3), DNN feature values of all individual units decoded from the measured brain activity patterns (Data Citations 4, 5), and decoding accuracies of individual units with their rankings among units (Data Citations 4, 5).

The fMRI dataset was originally collected for the study by Horikawa and Kamitani^6^ and comprises fMRI signals from five subjects measured while the subjects viewed sequences of natural images (image presentation experiment). This image presentation experiment had two sessions: a training image session and a test image session. Data from the training and test image sessions consist of fMRI responses to a total of 1,200 and 50 images respectively (“training” and “test” datasets).

The fMRI dataset was used to generate decoded DNN features for individual subjects. Using two types of DNN models, AlexNet^11^ and VGG19^12^, we first computed DNN feature values from the images presented in the fMRI experiments. We then trained a set of statistical linear regression models (decoders) to predict DNN feature values of presented images from visual cortical activity patterns in the training dataset. The trained decoders were then applied to the test dataset to produce decoded feature values for the 50 test images for all individual DNN units.

The “decodability” of the individual DNN units was then evaluated for individual subjects. For each DNN unit, a Pearson correlation coefficient was calculated between a sequence of decoded feature values and that of true feature values for the presented 50 test images. Then, the rankings of the decodability were calculated among a set of units within each DNN layer.

Our validation analysis showed that while the decodability varied considerably across units, the units were highly correlated across subjects for most DNN layers of the tested DNN models. This result indicates systematic differences in the representation of visual images between the DNN and the human brain.

To summarize, the present dataset contains a set of resources that is made use of for the DNN feature decoding and for further analyses. We hope that this dataset will offer opportunities to the neuroscience and computer science communities to develop new brain-DNN hybrid applications based on decoded features, and to facilitate comparative studies aimed at revealing the gap between the brain and DNNs.

## Methods

The data used in this study comes from a previous study performed in our laboratory^6^. According to the journal policy, we here provide a self-contained description of the subjects, datasets, and preprocessing of the MRI data for the main experiments to make it possible to understand and reproduce the experiments and analyses without referring to associated publications.

### Subjects

Five healthy subjects (one female and four males, aged between 23 and 38) participated in this study. All subjects had normal or corrected-to-normal visual acuity, and had substantial experience participating in fMRI experiments. All studies were performed with the written informed consent of the subjects, and were approved by the Ethics Committee of ATR.

### Stimuli

The stimuli consisted of sequences of natural images collected from an online image database ImageNet^13^ (2011, fall release). We first selected 200 representative object categories (synsets) from the database, and then randomly assigned them to 150 training and 50 test categories. Eight images were selected from each training category and one from each test category. The selected images were cropped to the center.

### Experimental design

The subjects participated in an image presentation experiment, a retinotopy experiment, and a functional localizer experiment. All visual stimuli were rear-projected onto a screen in the MRI scanner bore using a luminance-calibrated LCD projector. The image presentation experiment data were collected from each subject over multiple scanning sessions spanning approximately 2 months. On each experimental day, one consecutive session was conducted for a maximum of 2 h. Subjects were given adequate time for rest between runs (every 3–10 min), and were allowed to take a break or stop the experiment at any time.

The image presentation experiment consisted of two distinct types of sessions: training image sessions and test image sessions, comprising 24 and 35 separate runs (9 minutes 54 s for each run) respectively. Each run contained 55 stimulus blocks comprising 50 blocks with different images and 5 randomly interspersed repetition blocks where the same image as in the previous block was presented. In each stimulus block, an image (12 × 12 degrees of visual angle) was flashed at 1 Hz for 9 seconds (there was no inter-block interval). Images were presented at the center of the display with a central fixation spot. The color of the fixation spot changed from white to red for 0.5 s before each stimulus block began, to indicate the onset of the block. Extra 33-second and 6-second rest periods were added to the beginning and end of each run respectively. Subjects were instructed to maintain steady fixation on the fixation spot throughout each run, and performed a one-back repetition detection task on the images, responding with a button press for each repetition, to ensure that they maintained their attention on the presented images (mean task performance across five subjects: sensitivity = 0.930; specificity = 0.995). In the training image session, a total of 1,200 images from 150 categories (eight images from each category) were each presented once. In the test image session, a total of 50 images from 50 object categories (one image from each category) were presented 35 times each. The presentation order of the categories was randomized across runs.

The retinotopy experiment was performed following the conventional protocol^14,15^, using a rotating wedge and expanding ring of a flickering checkerboard. The data were used to delineate the borders between each visual cortical area, and to identify the retinotopic map (V1–V4) on the flattened cortical surfaces of individual subjects.

The functional localizer experiment was performed to identify the lateral occipital complex (LOC)^16^, fusiform face area (FFA)^17^, and parahippocampal place area (PPA)^18^ of each individual subject. The localizer experiment consisted of four to eight runs (varied across subjects), with each run containing 16 stimulus blocks. In this experiment, intact or scrambled images (12 × 12 degrees of visual angle) from face, object, house, and scene categories were presented at the center of the screen. Each of eight stimulus types (four categories × two conditions) was presented twice per run. Each stimulus block consisted of a 15-second intact or scrambled image presentation. The intact and scrambled stimulus blocks were presented successively (the order of the intact and scrambled stimulus blocks was randomized), followed by a 15-second rest period comprising a uniform gray background. Extra 33-second and 6-second rest periods were added to the beginning and end of each run, respectively. In each stimulus block, 20 different images of the same type were presented for 0.3 s, followed by an intervening blank screen of 0.45-second duration.

### MRI acquisition

MRI data were collected using a 3.0-Tesla Siemens MAGNETOM Trio A Tim scanner located at the ATR Brain Activity Imaging Center. An interleaved T2^∗^-weighted gradient-echo EPI scan was used to acquire the functional images covering the entire brain (image presentation and localizer experiments: TR, 3,000 ms; TE, 30 ms; flip angle, 80 deg; FOV, 192 × 192 mm; voxel size, 3 × 3 × 3 mm; slice gap, 0 mm; number of slices, 50) or the entire occipital lobe (retinotopy experiment: TR, 2,000 ms; TE, 30 ms; flip angle, 80 deg; FOV, 192 × 192 mm; voxel size, 3 × 3 × 3 mm; slice gap, 0 mm; number of slices, 30). T2-weighted turbo spin echo images were acquired as high-resolution anatomical images of the same slices used for the EPI (image presentation and localizer experiments: TR, 7,020 ms; TE, 69 ms; flip angle, 160 deg; FOV, 192 × 192 mm; voxel size, 0.75 × 0.75 × 3.0 mm; retinotopy experiment: TR, 6,000 ms; TE, 57 ms; flip angle, 160 deg; FOV, 192 × 192 mm; voxel size, 0.75 × 0.75 × 3.0 mm). T1-weighted magnetization-prepared rapid acquisition gradient-echo (MP-RAGE) fine-structural images of the entire head were also acquired (TR, 2,250 ms; TE, 3.06 ms; TI, 900 ms; flip angle, 9 deg; FOV, 256 × 256 mm; voxel size, 1.0 × 1.0 × 1.0 mm).

### MRI data preprocessing

The first 9-second of scans in experiments with a TR of 3 seconds (three volumes; image presentation and localizer experiments) and the first 8-second of scans in experiments with a TR of 2 s (four volumes; retinotopy experiment) were discarded from each run to avoid MRI scanner instability. The acquired fMRI data underwent three-dimensional motion correction using SPM5 (http://www.fil.ion.ucl.ac.uk/spm). The data were then coregistered to the within-session high-resolution anatomical image with the same slice dimensions and coordinates as the EPI, and subsequently to the whole-head high-resolution anatomical image. The coregistered data were then reinterpolated to 3 × 3 × 3 mm voxels.

For the data from the image presentation experiment, data samples were created by first regressing out nuisance parameters from each voxel amplitude for each run, including a constant baseline, a linear trend, and temporal components proportional to the six motion parameters calculated from the SPM motion correction procedure. The data were then despiked to reduce extreme values (beyond ± 3 SD for each run) and the voxel amplitudes were averaged within each 9-second stimulus block (three volumes) after shifting the data by 3 second (one volume) to compensate for hemodynamic delays.

### Region of interest (ROI) selection

V1, V2, V3, and V4 were delineated by the standard retinotopy experiment^14,15^. The data from the retinotopy experiment were transformed to Talairach coordinates and the visual cortical borders were delineated on flattened cortical surfaces using BrainVoyager QX (http://www.brainvoyager.com). The voxel coordinates around the gray-white matter boundary in V1–V4 were identified and transformed back into the original coordinates of the EPI images. The LOC, FFA, and PPA were identified using conventional functional localizers^16–18^. The data from the functional localizer experiment were analyzed using SPM5. The voxels showing significantly higher responses to intact object, face, or scene images than to corresponding scrambled images (two-sided *t*-test, uncorrected *P* < 0.05 or 0.01) were identified, and defined as LOC, FFA, and PPA, respectively. A contiguous region covering the LOC, FFA, and PPA was manually delineated on the flattened cortical surfaces, and this region was defined as the “higher visual cortex” (HVC). Voxels from V1–V4 and the HVC were combined to define the “visual cortex” (VC). In the regression analysis, voxels showing the highest correlation coefficient with the target variable in the training dataset were provided to decoders constructed for individual feature units (with a maximum of 500 voxels).

### Deep neural networks (DNNs)

We used the *Caffe* implementation of the AlexNet^11^ and VGG19^12^ deep neural network models (available from https://github.com/BVLC/caffe/), both of which were pre-trained with images in ImageNet^13^ to classify 1,000 object categories. The AlexNet consisted of five convolutional layers (conv1, conv2, conv3, conv4, and conv5) and three fully-connected layers (fc6, fc7, and fc8). The VGG19 model consisted of a total of sixteen convolutional layers (conv1_1, conv1_2, conv2_1, conv2_2, conv3_1, conv3_2, conv3_3, conv3_4, conv4_1, conv4_2, conv4_3, conv4_4, conv5_1, conv5_2, conv5_3, and conv5_4), and three fully-connected layers (fc6, fc7, and fc8). The outputs from the units in each of the DNN layers (immediately after convolutional or fully connected layers and before rectification) were used as target variables in the following feature decoding analysis.

### Deep neural network feature decoding

We used a set of linear regression models to construct multivoxel decoders to decode DNN feature values of a seen image from an fMRI activity pattern. For this purpose, we used a sparse linear regression (SLR) algorithm^19^ that could automatically select the voxels important for prediction. In our analysis, a single regression model (decoder) was constructed to predict feature values of a single DNN unit. In the following, we explain the regression model for a single DNN unit. We individually trained multiple models to predict feature values of all DNN units in the tested DNN layers and models.

Given an fMRI data sample **x**={*x*_1_,…,*x*_*d*_}^T^ comprising the activities of *d* voxels as input, the regression function can be expressed by

$$y\left(x\right)=\sum_{i=1}^{d}w_{i}x_{i}+w_{0},$$

where *x*_*i*_ is the fMRI amplitude of the voxel *i*, *w*_*i*_ is the weight of voxel *i*, and *w*_0_ is the bias. For simplicity, the bias *w*_0_ is included in the weight vector such that $w={\left\{w_{0},\ldots ,w_{d}\right\}}^{T}$. The dummy variable *x*_0_=1 is introduced into the data such that $x={\left\{x_{0},\ldots ,x_{d}\right\}}^{T}$. Using this regression function, we modelled the activity of a DNN unit as a target variable *t* explained by the regression function *y*(**x**) with additive Gaussian noise, as described by

t=y(x)+ε

where *ε* is a zero mean Gaussian random variable with noise precision *β*.

Given a training data set, SLR computes the weights for the regression function such that the regression function optimizes an objective function. To construct the objective function, we first express the likelihood function as

$$P\left(t|X,w,\beta \right)=\prod_{n=1}^{N}\frac{1}{{\left(2\pi \right)}^{1/2}}\beta^{1/2}\exp\left\{-\frac{1}{2}\beta {\left(t_{n}-w^{T}x_{n}\right)}^{2}\right\},$$

where *N* is the number of samples, **X** is an *N*×(*d* + 1) fMRI data matrix whose *n*th row is the *d* + 1-dimensional vector **x**_*n*_, and **t**={*t*_1, …, _*t*_*N*_}^T^ are the samples of a DNN unit.

To introduce sparsity into the weight estimation, we performed Bayesian parameter estimation and adopted the automatic relevance determination (ARD) prior^19^. We considered the estimation of the weight parameter **w** given the training data sets {**X**, **t**}. We assumed a Gaussian distribution prior for the weights **w** and non-informative priors for the weight precision parameters **α**={α_0_, … α_*d*_}^T^ and the noise precision parameter *β*, which are described as

$$P_{0}\left(w|\alpha \right)=\prod_{i=0}^{d}\frac{1}{{\left(2\pi \right)}^{1/2}}{\alpha_{i}}^{1/2}\exp\left\{-\frac{1}{2}\alpha_{i}{w_{i}}^{2}\right\},$$

$$P_{0}(\alpha )=\prod_{i=0}^{d}\frac{1}{\alpha_{i}},$$

$$P_{0}\left(\beta \right)=\frac{1}{\beta }.$$

In the Bayesian framework, we considered the joint probability distribution of all the estimated parameters, and the weights can be estimated by evaluating the following joint posterior probability of **w**:

$$P\left(w,\alpha ,\beta |X,t\right)=\frac{P\left(t,w,\alpha ,\beta |X\right)}{\int^{\;}dwd\alpha d\beta \;P\left(t,w,\alpha ,\beta |X\right)}=\frac{P\left(t|X,w,\beta \right)P_{0}\left(w|\alpha \right)P_{0}(\alpha )P_{0}(\beta )}{\int^{\;}dwd\alpha d\beta \;P\left(t,w,\alpha ,\beta |X\right)}.$$

Given that the evaluation of the joint posterior probability *P*(**w**, **α**, *β*|**X**, **t**) is analytically intractable, we approximated it using the variational Bayesian method^19–21^. While the results presented in this manuscript were obtained from the models with the ARD prior, qualitatively similar results were obtained using other regression models (e.g., ordinary least square regression model).

We trained linear regression models that decode feature values of individual feature units for seen images given fMRI samples in the training image session. For the test dataset, fMRI samples corresponding to the same images (35 samples for each of the 50 test images) were averaged across trials to increase the signal to noise ratio of the fMRI signals. Using the learned models, we decoded feature values of seen images from averaged fMRI samples. The feature decoding accuracy of each DNN unit was evaluated by the Pearson correlation coefficient between the true and decoded feature values of each feature unit. The estimated correlation coefficients (“decodability”) from individual subjects and their averages were ranked separately within each DNN layer and model. We assigned *nan* values to the decodability correlations and ranks of units not showing any responses (DNN signals) to images in the training or test datasets.

### Preferred images of individual units

We used the activation maximization technique to generate preferred images of individual units in each DNN layer^22–25^. The generation of preferred images starts with a random image and optimizes the image to maximally activate a target DNN unit by iteratively calculating how the image should be changed via backpropagation. This analysis was implemented using custom software written in MATLAB based on Python codes provided in a series of blog posts (Mordvintsev, A., Olah, C., Tyka, M., DeepDream—a code example for visualizing Neural Networks, https://github.com/google/deepdream, 2015; Øygard, A. M., Visualizing GoogLeNet Classes, https://github.com/auduno/deepdraw, 2015).

### Code availability

The code for the DNN feature decoding is available at https://github.com/KamitaniLab/GenericObjectDecoding. Both MATLAB and Python scripts are included in the repository. We also provide a Python API to download and extract data from Figshare (Data Citations 2, 4), and jupyter notebooks for example usage of the data at https://github.com/KamitaniLab/brain-decoding-datasets. In addition, Python scripts for generating preferred images of individual DNN units are also available at https://github.com/KamitaniLab/cnnpref. All code is available without any access restrictions.

## Data Records

### Experimental data

All the data produced from the MRI experiments are hosted at OpenNeuro (Data Citation 1). The dataset is based on the Brain Imaging Data Structure (BIDS)^26^. All MRI images are saved as NIfTI files.

The data repository contains five directories for the five subjects (*sub-01* to *sub-05*). Each directory comprises several subdirectories that include MRI data from a single scanning session. The *ses-anatomy* directory contains a defaced T1-weighted anatomical reference image for the individual subject, and the *ses-perceptionTraining^∗^* and *ses-perceptionTest^∗^* directories include fMRI images collected in the training and test image presentation experiments, respectively (Table 1). fMRI images from a single run are stored in a single 4-D NIfTI file. Each run is accompanied by a task event file, which describes experimental information such as the timing of trials, presented stimuli, and subject’s response times (Table 2). The session directories also contain a T2-weighted anatomical image obtained in the same session. Binary mask images for ROIs used in the analysis (see above) are placed in *sourcedata/ <subject> /anat* directories (Table 3).

In the task event files, stimuli are represented by a float number, *stimulus_id*, in which the integer part indicates the WordNet^27^ ID for the synset (category) and the decimal part indicates image ID. For example, *1518878.005958* represents image *5958* in synset *n01518878* (‘ostrich’). We do not include the stimulus images in the data repository because of licensing issues. A script to download the stimulus images is available at https://github.com/KamitaniLab/GenericObjectDecoding. Downloaded image files are named as *XXXX_YYYY.JPEG*, where *XXXX* and *YYYY* represents the WordNet ID and image ID respectively (e.g., *n01518878_5958.JPEG*).

### Preprocessed fMRI data

The preprocessed fMRI data are hosted at Figshare and Zenodo (Data Citations 2, 3). All data are saved as MATLAB (^∗^.mat) files. Each file contains data collected from one subject (Subject1-5.mat) as BrainDecoderToolbox2 data (https://github.com/KamitaniLab/BrainDecoderToolbox2). The data is composed of fMRI data (‘VoxelData’), stimulus labels (‘stimulus_id’), and experiment design information (‘DataType’ and ‘Run’). The fMRI data includes preprocessed BOLD signal values of voxels in ROIs that are used in the analysis (V1, V2, V3, V4, LOC, FFA, PPA, and HVC). Each row in the fMRI data array is within-block averaged signals. Signals during rest periods and one-back repetition blocks are excluded from the data. ‘stimulus_id’ is given by the same convention to Experimental Data (see above). ‘DataType’ represents the type of experiments; 1 and 2 represent training and test image presentation experiments, respectively. All the preprocessed fMRI data uploaded on Figshare (Data Citation 2) and Zenodo (Data Citation 3) are identical to each other.

### DNN features and decodability

The decoded features, true features, accuracy, and ranking by accuracy are available from Figshare and Zenodo (Data Citations 4, 5). All data files are saved as MATLAB (^∗^.mat) files and zipped by the DNN and the layer. The naming rules for the ^∗^.mat files and the size (shape) of the data in the file are summarized in Table 4. All DNN feature data uploaded on Figshare (Data Citation 4) and Zenodo (Data Citation 5) are identical to each other.

#### Decoded DNN features

The decoded features are saved to a file named in the manner ‘decoded–< *net* >–<*layer* >–<*subject_id* >–<*image_id* > .mat’, where <*net > *takes either “AlexNet” or “VGG19”, <*layer > *takes the layer name of the DNN, <*subject_id* > is the subject ID, and <*image_id* > is the ImageNet ID of the stimulus image. In the ^∗^.mat file, an array is saved with a shape the same as that of the output of the <*layer* > layer in the <*net* > DNN model (See Table 4). The ^∗^.mat files are zipped for each DNN and layer to ‘decodedDNN-decoded–<*net >*–<*layer>*.zip’ file and uploaded to Figshare and Zenodo (Data Citations 4, 5).

#### True DNN features

The true features are saved for each DNN model, layer, and image, and are named as ‘true–<*net*>–<*layer*>–<*image_id*>.mat’. Zipped files for each<*net*> and<*layer*> are uploaded to Figshare and Zenodo (Data Citations 4, 5).

#### Decoding accuracy (“decodability”)

The decoding accuracy for each DNN, layer, and subject are saved in a file named in the manner ‘accuracy–<*net*>–<*layer*>–<*subject_id*>.mat’. In addition, we also created files for accuracy averaged across subject, ‘accuracy-<*net*>-<*layer*>-Averaged.mat’. Zipped files for each<*net*> and<*layer*> are uploaded to Figshare and Zenodo (Data Citations 4, 5).

#### Decodability ranking

The ranking of feature units by accuracy is provided for each DNN, layer, and subject, and is provided in a ^∗^.mat file named as ‘rank–<*net*>–<*layer*>–<*subject_id*>.mat’. In addition, we also created a file containing the average ranking by subject, ‘rank–<*net*>–<*layer*>–Averaged.mat’. Zipped files for each<*net*> and<*layer*> are uploaded to Figshare and Zenodo (Data Citations 4, 5).

We also provide a Python API to download and extract data from Figshare (https://github.com/KamitaniLab/brain-decoding-datasets, see Usage Notes section).

## Technical Validation

To validate the quality of the dataset, we first performed feature decoding analysis to decode the DNN feature values from the fMRI activity patterns, and evaluated the decoding accuracy (“decodability”) of individual DNN units for each subject^6^. We then evaluated the consistency of the decodability between multiple subjects to demonstrate the replicability of the results across subjects.

In the feature decoding analysis, decoders were trained to decode DNN unit activities to input image sequences from visual cortical (VC) activities measured while the subjects viewed the same sequences of stimulus images from the training dataset (1200 samples). The decoders were individually trained for each unit in the convolutional (5 and 16 layers for AlexNet and VGG19 respectively) and fully-connected layers (3 layers for both of AlexNet and VGG19) of the DNN models (see Table 4 for the numbers of units in each layer). The trained decoders were then applied to an independent test dataset (50 samples) to evaluate the decodability of individual DNN units. The decodability of each DNN unit was evaluated by calculating a correlation coefficient (Pearson correlation) between a pair of feature value sequences from the DNN (true features) and the brain activity of individual subjects (decoded features).

**Figure 2:**
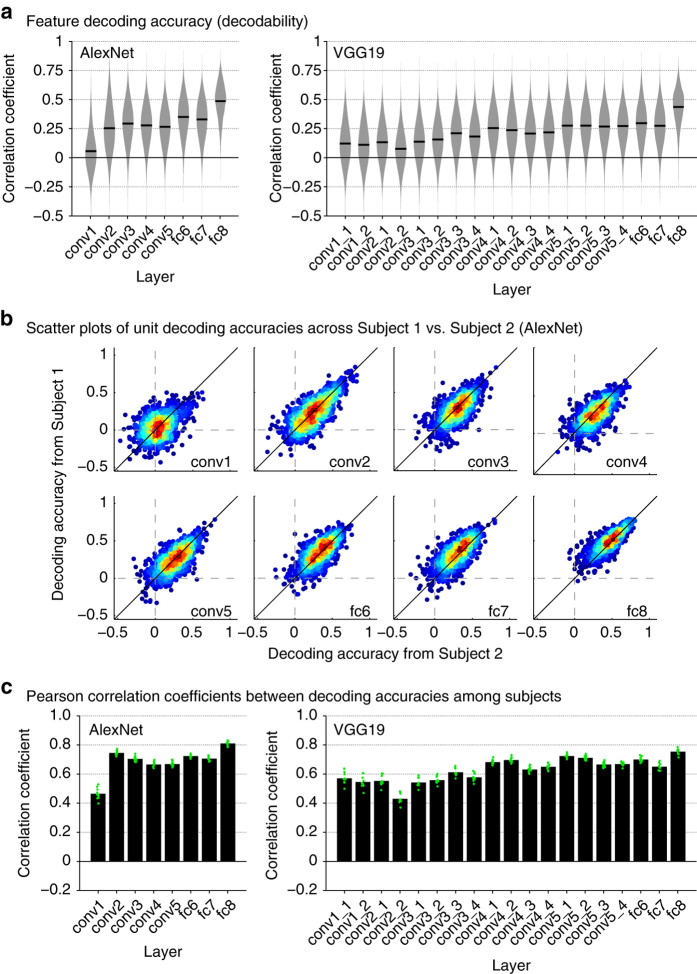
Evaluations of DNN feature decoding. (**a**) Violin plots of feature decoding accuracy for each DNN layer and model. Distributions of the decoding accuracies of all individual units in each DNN layer are shown (pooled across five subjects, predicted from VC). Black bars denote mean decoding accuracies averaged across all units and subjects. (**b**) Scatter plots of decoding accuracies of individual DNN units from two subjects (AlexNet, VC). Each dot denotes the decoding accuracies of each DNN unit estimated from Subject 1 (vertical axis) and Subject 2 (horizontal axis). The color of each dot indicates the density of the plotted dots. For visualization purpose, randomly selected subsets of units are shown with a maximum of 1000 units. (**c**) Mean correlation coefficients between decoding accuracies of DNN units from different subjects (VC). Pearson correlation coefficients between decoding accuracies of individual DNN units obtained from different subjects were calculated for all pairs of subjects (10 pairs from 5 subjects). Each dot denotes the correlation coefficients for each pair of subjects.

The obtained decodabilities of individual units were further examined for each DNN layer and model, and were compared across subjects. Figure 2a shows the distributions of the feature decoding accuracies evaluated for each individual layer of each DNN model (AlexNet and VGG19), in which the decoding accuracy largely varied across units, layers, and models. To assess the degree of consistency of decodability across subjects, we evaluated the unit-by-unit similarity of the decodability between multiple subjects. Figure 2b shows example scatter plots of feature decoding accuracies from two subjects. The decodability of individual units from the two subjects is densely distributed along the diagonal axis for most layers, showing positive correlations between the two subjects. Figure 2c shows the mean correlation coefficients across all pair combinations of the five subjects. The decodability shows positive correlation coefficients between subjects for all layers of the each of the two DNN models. These results suggest that the feature decoding from the brain can produce replicable results and that the decodability was highly consistent across subjects even at the unit level.

Taken together, our analyses support the quality of the present dataset as the data showed replicable and consistent results from multiple subjects. The fMRI data made it possible to decode DNN feature values from brain activity patterns, and the estimated decodability was highly consistent across subjects. Thus, free availability of the present dataset provides an opportunity for it to be utilized for various purposes, including the feature selection in neural encoding and decoding analyses^4,8,10^, as well as further applications involving combining the decoded features with deep neural network technology^6,9^.

## Usage Notes

The experimental data can be downloaded from OpenNeuro (Data Citation 1). To perform DNN feature decoding, the fMRI data need to be preprocessed as described in the Methods section. The head motion correction, functional-anatomical registration in individual anatomical space, and resampling, can be conducted with SPM, while the further preprocessing, including regressing-out of nuisance parameters, reduction of extreme values, shifting of data, and within-block averaging can be conducted with Brain Decoder Toolbox 2 (https://github.com/KamitaniLab/BrainDecoderToolbox2). The preprocessed fMRI data are available on Figshare and Zenodo (Data Citations 2, 3)

The DNN feature decoding analysis can be performed with scripts available at https://github.com/KamitaniLab/GenericObjectDecoding (*analysis_FeaturePredicion.m* for MATLAB and *analysis_FeaturePrediciton.py* for Python). The scripts train the feature decoding models with fMRI data in the training image presentation experiments, and predict DNN features from fMRI data in the test image presentation experiments. To allow the data to be read by the scripts, the fMRI data must be saved in Brain Decoder Toolbox 2 format.

The decoded DNN features are available on Figshare and Zenodo (Data Citations 4, 5). We provide a Python API for downloading and extracting data from Figshare (https://github.com/KamitaniLab/brain-decoding-datasets). The repository also includes a jupyter notebook that replicates results in Fig. 2.

## Additional information

**How to cite this article**: Horikawa, T. *et al*. Characterization of deep neural network features by decodability from human brain activity. *Sci. Data*. 6:190012 https://doi.org/10.1038/sdata.2019.12 (2019).

**Publisher’s note**: Springer Nature remains neutral with regard to jurisdictional claims in published maps and institutional affiliations.

## Supplementary Material



## Figures and Tables

**Table 1:** Summary of the experimental data.

Subject	Experiment	Session	# runs
Subject 1 (*sub-01*)	Training image (*ses-perceptionTraining*)	1	10
		2	10
		3	4
	Test image(*ses-perceptionTest*)	1	10
		2	10
		3	5
		4	10
Subject 2 (*sub-02*)	Training image(*ses-perceptionTraining*)	1	10
		2	10
		3	4
	Test image(*ses-perceptionTest*)	1	10
		2	10
		3	10
		4	5
Subject 3 (*sub-03*)	Training image(*ses-perceptionTraining*)	1	8
		2	8
		3	8
	Test image(*ses-perceptionTest*)	1	8
		2	9
		3	8
		4	6
		5	4
Subject 4 (*sub-04*)	Training image(*ses-perceptionTraining*)	1	8
		2	8
		3	8
	Test image(*ses-perceptionTest*)	1	9
		2	9
		3	9
		4	8
Subject 5 (*sub-05*)	Training image(*ses-perceptionTraining*)	1	8
		2	4
		3	6
		4	3
		5	3
	Test image(*ses-perceptionTest*)	1	7
		2	7
		3	5
		4	4
		5	5
		6	7

**Table 2:** Columns in task event files for the image presentation experiments.

Column	Description
onset	Onset time of the event (sec)
duration	Duration of the event (sec)
trial_no	Trial number
event_type	Type of the event (*rest* or *stimulus*)
stim_id	Stimulus ID
response_time	Subject’s response time (sec; elapsed time from the beginning of the run)

**Table 3:** ROI mask images included in the dataset.

File name	ROI
sub-*_mask_LH_V1.nii.gz	Left V1
sub-*_mask_RH_V1.nii.gz	Right V1
sub-*_mask_LH_V2.nii.gz	Left V2
sub-*_mask_RH_V2.nii.gz	Right V2
sub-*_mask_LH_V3.nii.gz	Left V3
sub-*_mask_RH_V3.nii.gz	Right V3
sub-*_mask_LH_hV4.nii.gz	Left V4
sub-*_mask_RH_hV4.nii.gz	Right V4
sub-*_mask_LH_LOC.nii.gz	Left LOC
sub-*_mask_RH_LOC.nii.gz	Right LOC
sub-*_mask_LH_FFA.nii.gz	Left FFA
sub-*_mask_RH_FFA.nii.gz	Right FFA
sub-*_mask_LH_PPA.nii.gz	Left PPA
sub-*_mask_RH_PPA.nii.gz	Right PPA
sub-*_mask_LH_HVC.nii.gz	Left higher visual cortex (HVC)
sub-*_mask_RH_HVC.nii.gz	Right higher visual cortex (HVC)

**Table 4:** Summary of the DNN feature and decodability datasets.

<*data_type*>	<*subject_id*>	<*image_id*>	<*net*>	<*layer*>	Data size
accuracydecodedranktrue	S1S2S3S4S5Averaged (only for accuracy and rank)	ImageNet ID for 50 stimulus images in the format n*****_****.(only for decoded and true)	AlexNet	conv1	55 × 55 × 96
				conv2	27 × 27 × 256
				conv3	13 × 13 × 384
				conv4	13 × 13 × 384
				conv5	13 × 13 × 256
				fc6	1 × 1 × 4096
				fc7	1 × 1 × 4096
				fc8	1 × 1 × 1000
			VGG19	conv1_1	224 × 224 × 64
				conv1_2	224 × 224 × 64
				conv2_1	112 × 112 × 128
				conv2_2	112 × 112 × 128
				conv3_1	56 × 56 × 256
				conv3_2	56 × 56 × 256
				conv3_3	56 × 56 × 256
				conv3_4	56 × 56 × 256
				conv4_1	28 × 28 × 512
				conv4_2	28 × 28 × 512
				conv4_3	28 × 28 × 512
				conv4_4	28 × 28 × 512
				conv5_1	14 × 14 × 512
				conv5_2	14 × 14 × 512
				conv5_3	14 × 14 × 512
				conv5_4	14 × 14 × 512
				fc6	1 × 1 × 4096
				fc7	1 × 1 × 4096
				fc8	1 × 1 × 1000
The data files in Figshare and Zenodo (Data Citations 4, 5) are named as “<*data_type*>–<*net*>–<*layer*>–<*subject_id*>–*<image_id*>.mat”. A list of each component and the size (shape) of the data are shown.					
